# Theoretical Simulation of Output Characteristics of an RTD-Fluxgate Sensor Under Sawtooth Wave Excitation

**DOI:** 10.3390/mi16040388

**Published:** 2025-03-28

**Authors:** Haibo Guo, Na Pang, Xu Hu, Rui Wang, Guo Li, Fei Li

**Affiliations:** College of Computer Science and Technology, Beihua University, No. 3999 East Binjiang Road, Jilin 132013, China; ghb@beihua.edu.cn (H.G.); hx@beihua.edu.cn (X.H.); wangrui@beihua.edu.cn (R.W.); lg@beihua.edu.cn (G.L.); lifei@beihua.edu.cn (F.L.)

**Keywords:** excitation waveform, RTD-fluxgate, sensitivity, sawtooth wave

## Abstract

With the widespread application of RTD-fluxgate sensors in UAV aeromagnetic measurements, improving sensor sensitivity is essential for aeromagnetic gradient detection. The excitation waveform is one of the key factors affecting sensitivity. Under sinusoidal excitation, the output model shows poor linearity, and the time-difference expression needs to consider coercivity. Additionally, when triangular and trapezoidal waves are used, sensitivity improvement is limited. To address these issues, this paper proposed using a sawtooth wave as the excitation waveform for RTD-fluxgate sensors. The expressions for output time difference ΔT and sensitivity S were derived, and the sensor’s output characteristics under different excitations were compared. It was found that the time-difference expression under sawtooth wave excitation was independent of coercivity. The simulation results showed that under identical frequency and amplitude conditions, the time difference ΔT produced by sawtooth wave excitation was 2 times that of the triangular wave and 3.3 times that of the trapezoidal wave, significantly enhancing sensitivity. This excitation waveform offers advantages, providing new technical support for UAV aeromagnetic gradient detection and demonstrating broad application potential.

## 1. Introduction

In recent years, unmanned aerial vehicle (UAV) aeromagnetic measurements have emerged as a hotspot in research as a novel aeromagnetic technology [1]. In complex terrain areas in China where traditional aeromagnetic exploration cannot be conducted, such as regions with high-altitude differences, intricate ravines, and dense water systems, UAV technology provides an innovative solution. Thanks to its compact size, highly intelligent operation, flexible transport and control capabilities, and the ability to conduct multiple flights, UAVs can maintain low-altitude flights in these rugged areas to perform precise aeromagnetic measurement tasks [2]. Meanwhile, fluxgate sensors are characterized by their small size, light weight, and low production costs, aligning well with the lightweight measurement requirements of UAVs as a carrying platform [3]. Traditional even-harmonic fluxgate sensors are limited by odd-harmonic interference, structural symmetry requirements, and manufacturing processes, resulting in bottlenecks in sensitivity and resolution improvement. Additionally, the complexity and power consumption of detection circuits remain difficult to reduce. In contrast, the residence time difference (RTD)-fluxgate sensor measures the magnetic field using the bidirectional magnetic saturation time difference of a single-axis magnetic core, thereby avoiding the inherent issues of even-harmonic fluxgate sensors. Its structure is more simplified, and its detection accuracy has reached the highest level of existing even-harmonic fluxgate sensors, with further improvement potential. The time-difference quantization counting method reduces circuit complexity, enhances anti-interference capability, lowers inherent noise, and decreases power consumption. Moreover, it enables vector measurement, demonstrating significant advantages in high-precision magnetic gradient measurements. In recent years, RTD-fluxgate sensors have garnered attention both domestically and internationally in the fields of defense and geomagnetic exploration [4,5,6,7,8,9,10].

In 2003, a research project on “time-difference fluxgate sensors” funded by the U.S. Navy investigated the theory of time-difference measurement [11]. In 2005, Italian researchers, including Bruno Andò, studied sensitive units and completed the development of a time-difference fluxgate sensor with a micro-wire structure [12]. In 2012, researchers from Jilin University, led by Lu Hao, conducted a detailed analysis of the performance of RTD-fluxgate sensors when using sinusoidal and triangular waves as excitation waveforms [13]. In 2014, Jilin University’s Pang Na and colleagues suggested that trapezoidal wave excitation could improve the performance of RTD-fluxgate sensors and established a simulation model for these sensors [14,15]. In 2023, Bruno Andò and others from Italy established a model for a coupled time-difference fluxgate sensor system [16]. In summary, although extensive research has been conducted both domestically and internationally on RTD-fluxgate sensors, there is insufficient analysis of the output characteristics under sawtooth wave excitation. Currently, when sinusoidal waves are used for excitation, the sensor’s output model exhibits complex nonlinear relationships, and the time-difference expression is influenced by coercivity. While triangular and trapezoidal wave excitations do not require the consideration of coercivity in the time-difference expression, the sensitivity of the sensor still needs improvement. Therefore, this paper adopts a sawtooth wave as the excitation waveform for the sensor, resulting in a simpler time-difference formula that is unaffected by coercivity and can significantly enhance the sensor’s sensitivity under the same amplitude and frequency conditions.

## 2. The Research on the Output Model of the RTD-Fluxgate Sensor

### 2.1. The Working Principle of the RTD-Fluxgate Sensor

**Figure 1:**
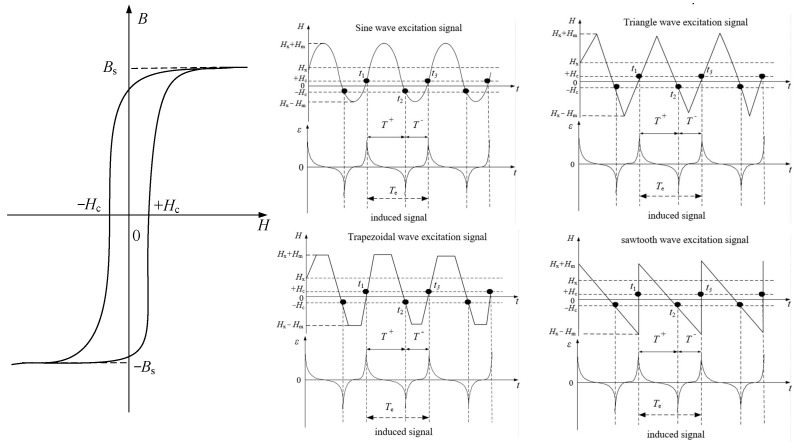
The schematic diagram of the working principle of the RTD-fluxgate sensor under different waveform excitations.

The RTD-fluxgate sensor primarily consists of a sensing unit and a detection circuit [17]. It measures the magnetic field by utilizing the relationship between the residence time in the positive and negative saturation states of the magnetized core and the magnetic field being measured [18]. The detection principle of the RTD-fluxgate sensor under different waveform excitations is shown in Figure 1. When the measured magnetic field ${\;H}_{x}$≠ 0, the time interval $T^{+}$ between the positive and negative pulses of the induced voltage output by the sensing unit and the time interval $T^{-}$ between the negative and positive pulses are different. By detecting the bidirectional magnetic saturation time difference ΔT between the positive and negative pulses of the induced voltage, the magnetic field ${\;H}_{x}$ is measured [19,20]. The relationship between the time difference ΔT and the measured magnetic field ${\;H}_{x}\;$ is related to the waveform of the excitation magnetic field ${\;H}_{e}(t)$. The output characteristics of the sensor vary with different excitation waveforms [13]. Therefore, it is necessary to study the sensor’s output model under different excitation waveforms.

### 2.2. Output Model Under Sine Wave Excitation

When using a sine wave magnetic field for excitation, the amplitude of the excitation magnetic field is assumed to be ${\;H}_{m}\;(A/m)$, the period is $T_{e}$ (s), and the frequency is f (Hz), In one period, *T* = 1/f,  θ is the phase angle, and the expression is given by Equation (1):

(1) $$H_{e}(t)=H_{m}sin(2\pi ft+\theta )$$

When the total magnetic field strength *H* reaches the positive and negative saturation states of the magnetic core at $t_{1}$, $t_{2}$, and $t_{3}$, the relationship between the excitation magnetic field $H_{e}(t)$, the measured magnetic field $H_{x}$, and the coercive force $H_{c}$ is given by Equation (2):

(2) $$\left\{\begin{array}{c} t_{1}:H_{x}+H_{m}\sin\left(2\pi ft_{1}+\theta \right)=H_{c}\;\;\;\;\;\;\;\;\;\;\;\;\;\;\;\;\;\;\;\;\;\\ t_{2}:H_{x}-H_{m}\sin\left[2\pi f\left(t_{2}-\frac{T_{e}}{2}\right)+\theta \right]=-H_{c}\\ t_{3}:t_{3}=t_{1}+T_{e}\;\;\;\;\;\;\;\;\;\;\;\;\;\;\;\;\;\;\;\;\;\;\;\;\;\;\;\;\;\;\;\;\;\;\;\;\;\;\;\;\;\;\;\;\;\;\;\;\;\;\;\; \end{array}\right.$$

From Equation (2), we derive:

(3) $$\left\{\begin{array}{c} t_{1}=\frac{1}{2\pi f}\left[\arcsin\left(\frac{H_{c}-H_{x}}{H_{m}}\right)-\theta \right]\;\;\;\;\;\;\;\;\;\;\\ t_{2}=\frac{1}{2\pi f}\left[\arcsin\left(\frac{H_{c}+H_{x}}{H_{m}}\right)-\theta \right]+\frac{T_{e}}{2}\\ t_{3}=\frac{1}{2\pi f}\left[\arcsin\left(\frac{H_{c}-H_{x}}{H_{m}}\right)-\theta \right]+T_{e} \end{array}\right.$$

The time intervals between the pulses in one period $T_{e}$ are given by:

(4) $$\left\{\begin{array}{c} T^{+}=t_{2}-t_{1}\;\;\;\;\;\;\;\;\;\;\;\;\;\;\;\;\;\;\;\;\;\\ =\frac{1}{2\pi f}\left[\arcsin\left(\frac{H_{c}+H_{x}}{H_{m}}\right)-\arcsin\left(\frac{H_{c}-H_{x}}{H_{m}}\right)\right]+\frac{T_{e}}{2}\;\;\;\;\\ T^{-}=t_{3}-t_{2}\;\;\;\;\;\;\;\;\;\;\;\;\;\;\;\;\;\;\;\;\;\\ =\frac{1}{2\pi f}\left[\arcsin\left(\frac{H_{c}-H_{x}}{H_{m}}\right)-\arcsin\left(\frac{H_{c}+H_{x}}{H_{m}}\right)\right]+\frac{T_{e}}{2}\;\;\;\; \end{array}\right.$$

Under sinusoidal magnetic field excitation, the relationship between the output time difference ΔT of the RTD-fluxgate sensor and the measured magnetic field $H_{x}$ is given by Equation (5):

(5) $$\Delta T=T^{+}-T^{-}=\frac{1}{\pi f}\left[arcsin\left(\frac{H_{c}+H_{x}}{H_{m}}\right)-arcsin\left(\frac{H_{c}-H_{x}}{H_{m}}\right)\right]$$

**Figure 2:**
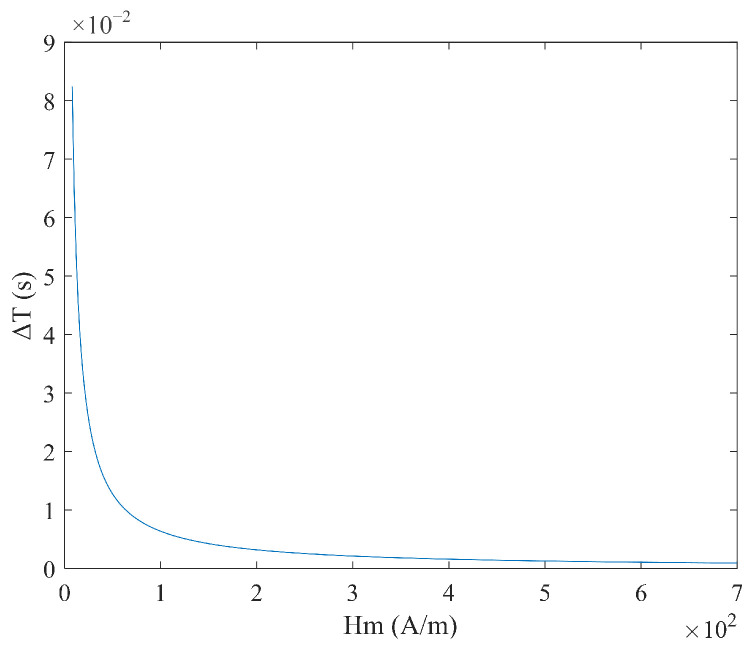
Relationship between time difference ΔT and excitation amplitude $H_{m}$ under sine wave excitation.

**Figure 3:**
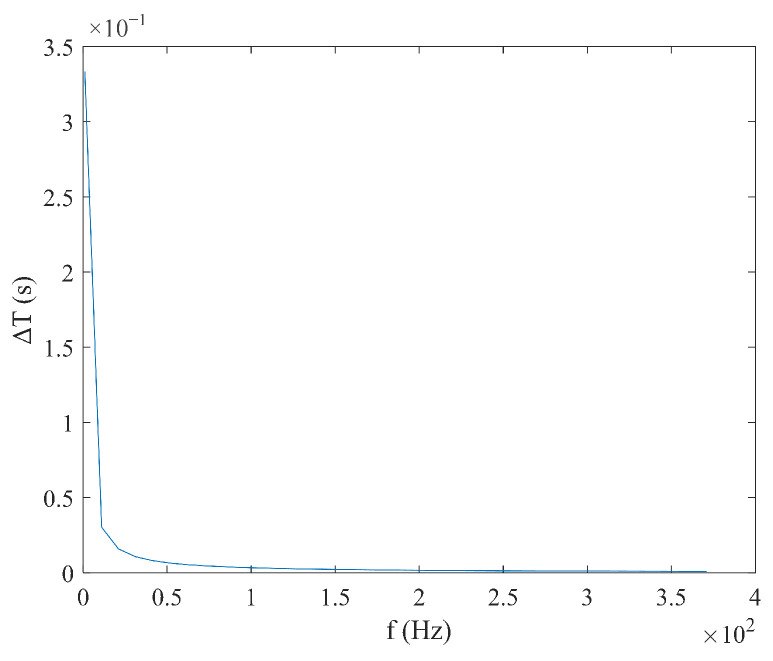
Relationship between time difference ΔT and frequency f under sine wave excitation.

From Equation (5), it is evident that the time difference ΔT expression is complex. It depends not only on the excitation amplitude $H_{m}$ and frequency f but also on the magnetic core coercive force $H_{c}$. When the measured magnetic field $H_{x}$ and coercive force ${\;H}_{c}$ are constant, the relationship between ΔT and the excitation amplitude $H_{m}$, and the frequency is shown in Figure 2 and Figure 3. As the excitation amplitude $H_{m}$ and frequency f decrease, the time difference ΔT increases. According to the sensitivity calculation the formula $S=\frac{\partial \Delta T}{\partial H_{x}}$, the sensitivity S under sine wave magnetic field excitation is expressed as:

(6) $$S=\frac{1}{\pi H_{m}f}\left(\frac{1}{\sqrt{1-{\left(\frac{H_{c}+H_{x}}{H_{m}}\right)}^{2}}}+\frac{1}{\sqrt{1-{\left(\frac{H_{c}-H_{x}}{H_{m}}\right)}^{2}}}\right)$$

**Figure 4:**
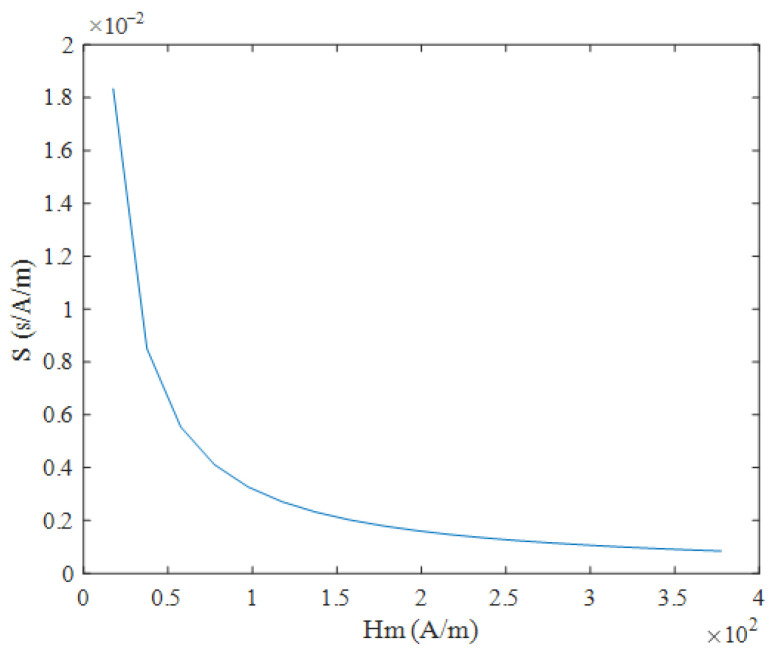
Relationship between sensitivity S and excitation amplitude $H_{m}$ under sine wave excitation.

**Figure 5:**
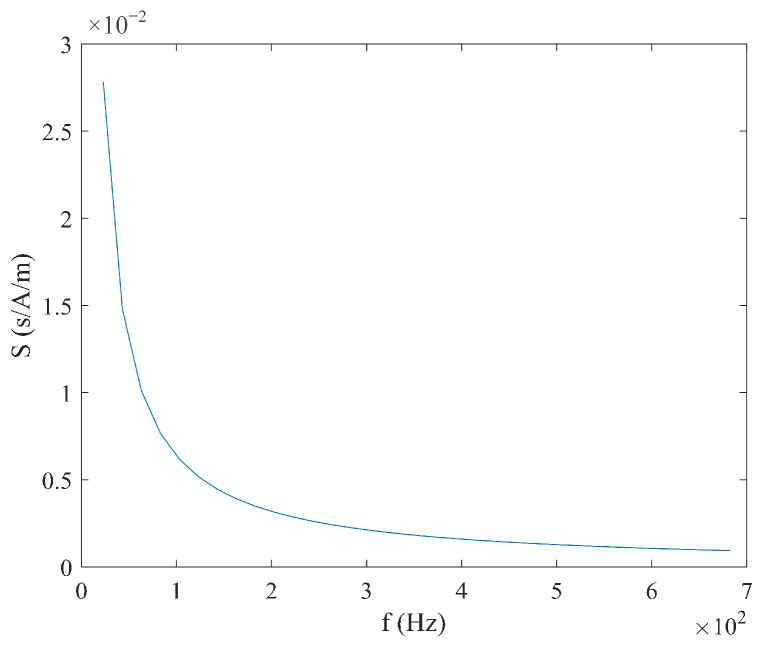
Relationship between sensitivity S and frequency f under sine wave excitation.

From Equation (6), it can be seen that the sensitivity S varies with the measured magnetic field $H_{x}$, and there is no linear relationship between the time difference ΔT and the measured magnetic field $H_{x}$. When the measured magnetic field $H_{x}$ and coercive force $H_{c}$ are constant, the relationship between sensitivity S, excitation amplitude${\;H}_{m}$, and frequency f is shown in Figure 4 and Figure 5. It can be observed that as the excitation amplitude $H_{m}$ and frequency f decrease, the sensitivity S increases.

### 2.3. Output Model Under Triangular Wave Excitation

When using a triangular wave magnetic field for excitation, the amplitude of the excitation magnetic field is assumed to be${\;\;H}_{m}$, the period is $T_{e}$, and the frequency is f. The slope of the sloping edge is ±a, and *N* is the number of periods. The expression is given by Equation (7):

(7) $$H_{e}\left(t\right)=\left\{\begin{array}{c} at\;\;\;\;NT_{e}-\frac{T_{e}}{4}<t<NT_{e}+\frac{T_{e}}{4}\;\;\;\;\;\;\\ -a\left(t-\frac{T_{e}}{2}\right)\;\;\;\;NT_{e}+\frac{T_{e}}{4}<t<NT_{e}+\frac{3T_{e}}{4} \end{array}\right.\;(N=0,1,2\ldots )$$

When the total magnetic field strength *H* reaches the saturation states of the magnetic core at $t_{1}$, $t_{2}$, and $t_{3}$, the time intervals between the pulses in one period $\;T_{e}$ are given by Equation (8):

(8) $$\left\{\begin{array}{c} T^{+}=t_{2}-t_{1}=\frac{2H_{x}}{a}+\frac{T_{e}}{2}\\ T^{-}=t_{3}-t_{2}=-\frac{2H_{x}}{a}+\frac{T_{e}}{2} \end{array}\right.$$

Under triangular magnetic field excitation, the relationship between the output time difference ΔT of the RTD-fluxgate sensor and the measured magnetic field${\;H}_{x}$ is given by Equation (9):

(9) $$\Delta T=T^{+}-T^{-}=\frac{4H_{x}}{a}=\frac{4H_{x}}{4H_{m}f}=\frac{H_{x}}{H_{m}f}$$

The expression for the sensitivity S of the RTD-fluxgate sensor is as follows:

(10) $$S=\frac{\partial \Delta T}{\partial H_{x}}=\frac{4}{a}=\frac{1}{H_{m}f}$$

**Figure 6:**
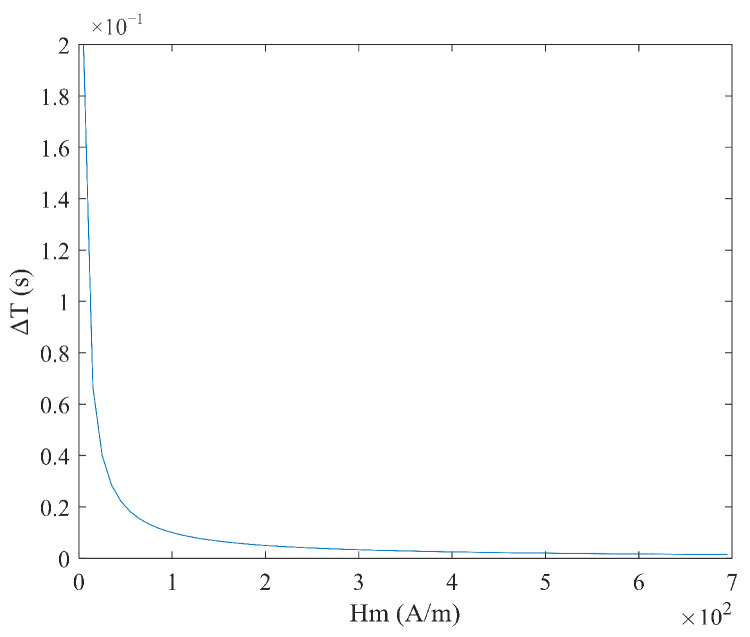
Relationship between time difference ΔT and excitation amplitude $H_{m}$ under triangular wave excitation.

**Figure 7:**
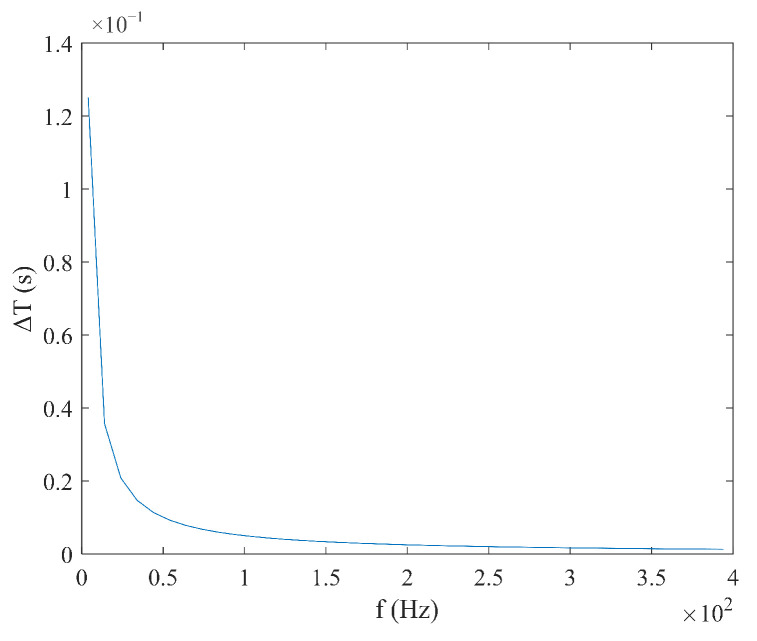
Relationship between time difference ΔT and frequency f under triangular wave excitation.

**Figure 8:**
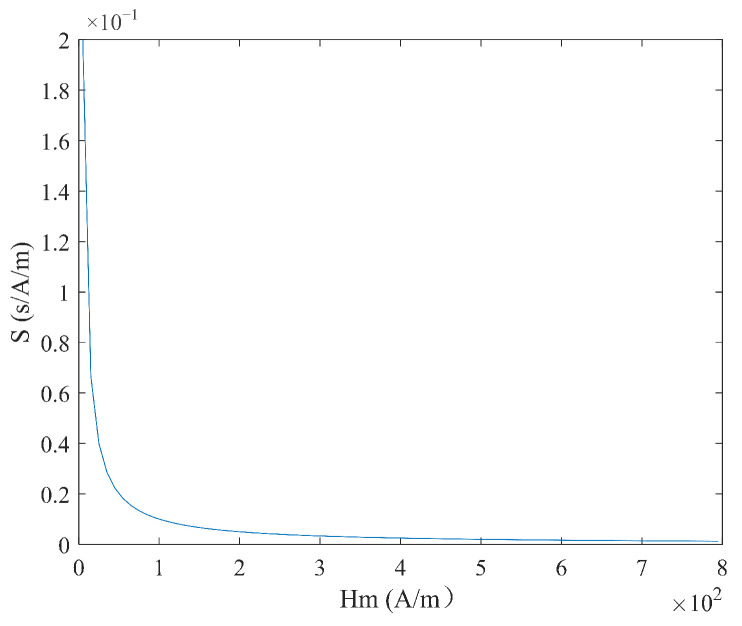
Relationship between sensitivity S and excitation amplitude$\;{\;H}_{m}$ under triangular wave excitation.

**Figure 9:**
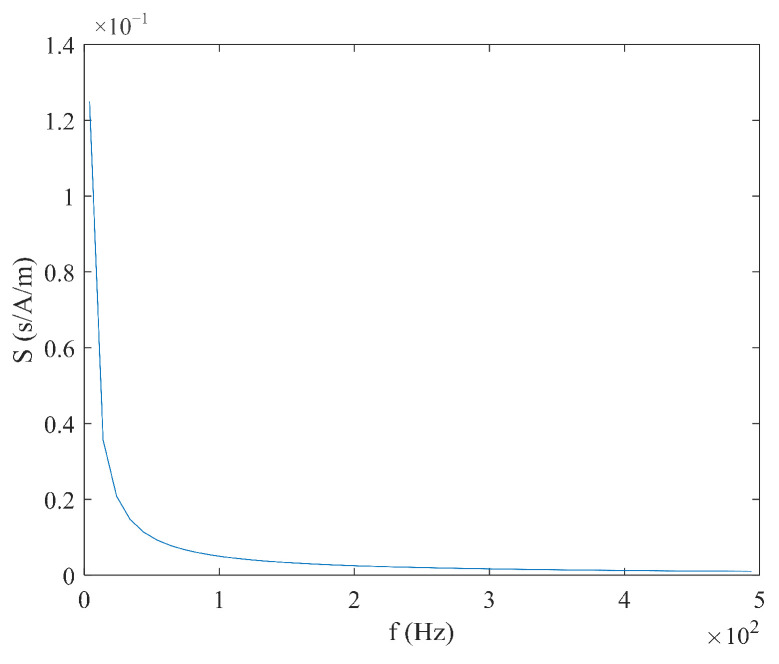
Relationship between sensitivity S and frequency f under triangular wave excitation.

According to Equation (9), under triangular magnetic field excitation, the size of coercivity $H_{c}$ does not need to be considered. When the measured magnetic field $H_{x}$ is constant, the time difference ΔT has a linear relationship with${\;\;H}_{x}$, and the expression is simple. Under these conditions, ΔT is inversely proportional to the excitation amplitude $H_{m}$ and frequency f, as shown in Figure 6 and Figure 7. According to Equation (10), the sensitivity S under triangular wave magnetic field excitation is independent of the measured magnetic field $H_{x}$ and is determined solely by the excitation amplitude $H_{m}$ and the frequency f. As shown in Figure 8 and Figure 9, sensitivity S is inversely proportional to both the excitation amplitude ${\;H}_{m}$ and frequency f.

### 2.4. Output Model Under Trapezoidal Wave Excitation

When using a trapezoidal wave magnetic field for excitation, assuming the total time for the sloping edges in one period is $\;t^{\prime }$, the stable time is $t^{\prime \prime }$, and the period is $T_{e}\;(s)$, then $t^{\prime }$ + $t^{\prime \prime }$ = $T_{e}$. The slope of the sloping edge is ±a, and the stable amplitude is $\pm {\;H}_{m}$(A/m). *N* is the number of periods. The expression is given by Equation (11):

(11) $$H_{e}\left(t\right)=\left\{\begin{array}{c} at\;\;\;\;NT_{e}-\frac{t^{\prime }}{4}<t<NT_{e}+\frac{t^{\prime }}{4}\;\;\;\;\;\\ H_{m}\;\;\;\;NT_{e}+\frac{t^{\prime }}{4}<t<NT_{e}+\frac{T_{e}}{2}-\frac{t^{\prime }}{4}\;\;\\ -at\;\;\;\;NT_{e}+\frac{T_{e}}{2}-\frac{t^{\prime }}{4}<t<NT_{e}+\frac{T_{e}}{2}+\frac{t^{\prime }}{4}\\ -H_{m}\;\;\;\;NT_{e}+\frac{T_{e}}{2}+\frac{t^{\prime }}{4}<t<NT_{e}+T_{e}-\frac{t^{\prime }}{4} \end{array}\right.\;(N=0,1,2\ldots )$$

When the total magnetic field strength reaches the coercive forces${\;H}_{c}$ and −${\;H}_{c}$, the moments of positive and negative saturation of the magnetic core are $t_{1}$, $t_{2}$, and $t_{3}$. The relationships between these magnetic fields can be expressed as:

(12) $$\left\{\begin{array}{c} t_{1}:H_{x}+at_{1}=H_{c}\;\;\;\;\;\\ t_{2}:H_{x}-a\left(t_{2}-\frac{T_{e}}{2}\right)=-H_{c}\\ t_{3}:t_{3}=t_{1}+T_{e}\;\;\;\;\;\;\;\; \end{array}\right.$$

From Equation (12), it can be derived that:

(13) $$\left\{\begin{array}{c} t_{1}=\frac{H_{c}-H_{x}}{a}\;\;\;\;\\ t_{2}=\frac{H_{c}+H_{x}}{a}+\frac{T_{e}}{2}\\ t_{3}=t_{1}+T_{e}\;\;\;\;\;\; \end{array}\right.$$

When the total magnetic field strength *H* reaches the saturation states of the magnetic core at $t_{1}$, $t_{2}$, and $t_{3}$, the time intervals between the pulses within one period $T_{e}$ are given by Equation (14):

(14) $$\left\{\begin{array}{c} T^{+}=t_{2}-t_{1}=\frac{2H_{x}}{a}+\frac{T_{e}}{2}\;\\ T^{-}=t_{3}-t_{2}=-\frac{2H_{x}}{a}+\frac{T_{e}}{2} \end{array}\right.$$

Under trapezoidal magnetic field excitation, the relationship between the output time difference ΔT of the RTD-fluxgate sensor and the measured magnetic field $H_{x}$ is given by Equation (15):

(15) $$\Delta T=T^{+}-T^{-}=\frac{4H_{x}}{a}=\frac{4H_{x}t^{\prime }}{4H_{m}}=\frac{H_{x}t^{\prime }}{H_{m}}$$

The expression for the sensitivity S of the RTD-fluxgate sensor is as follows:

(16) $$S=\frac{\partial \Delta T}{\partial H_{x}}=\frac{4}{a}=\frac{t^{\prime }}{H_{m}}$$

**Figure 10:**
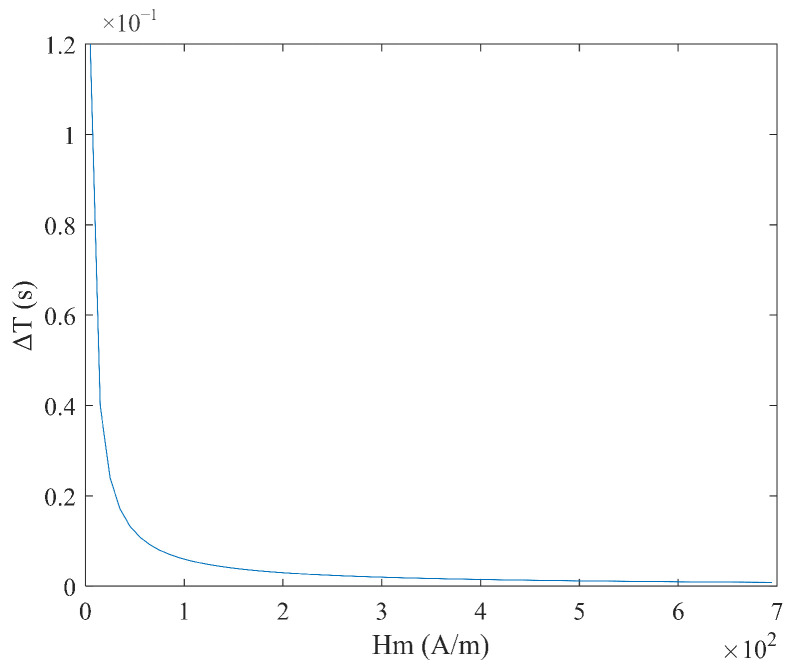
Relationship between time difference ΔT and excitation amplitude ${\;H}_{m}$ under trapezoidal wave excitation.

**Figure 11:**
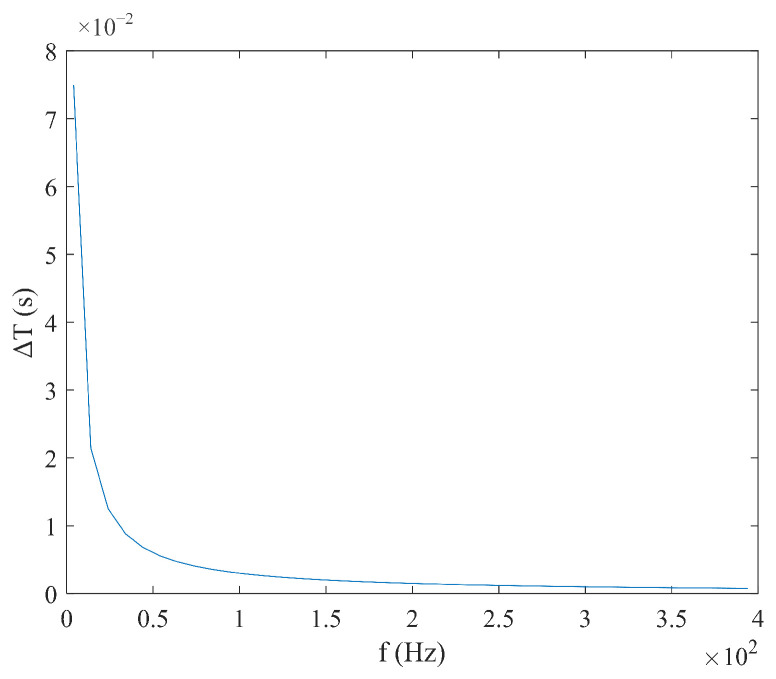
Relationship between time difference ΔT and frequency f under trapezoidal wave excitation.

**Figure 12:**
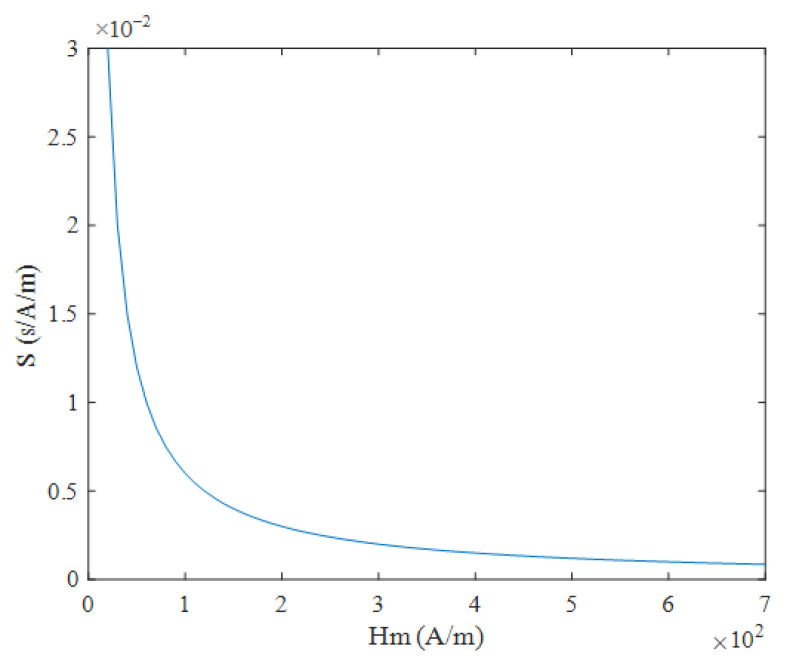
Relationship between sensitivity S and excitation amplitude $H_{m}$ under trapezoidal wave excitation.

**Figure 13:**
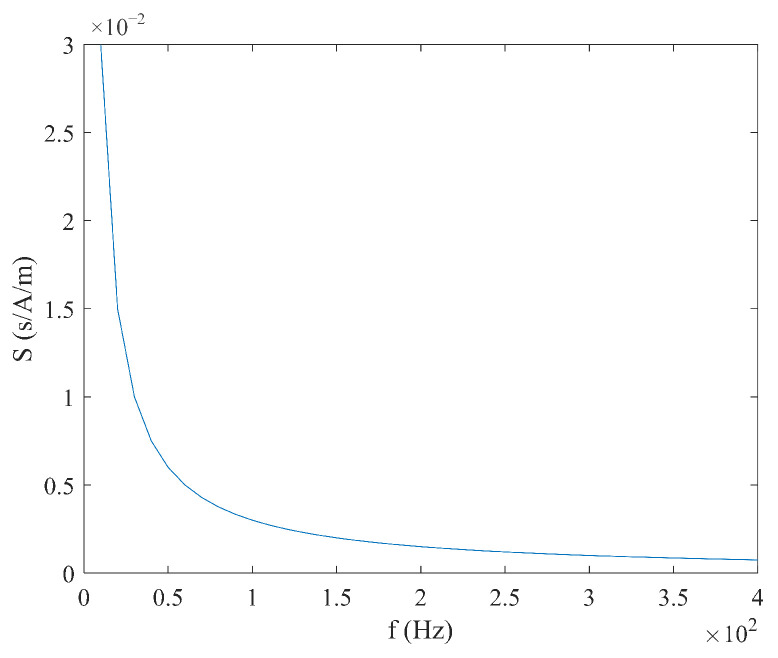
Relationship between sensitivity S and frequency f under trapezoidal wave excitation.

According to Equations (15) and (16), when the slope of the trapezoidal waveform remains constant, the sensitivity S of the RTD-fluxgate sensor is constant, and the time difference ΔT has a linear relationship with the measured magnetic field $H_{x}$. As shown in Figure 10 and Figure 11, when the ratio of the total time of the sloped edges to the period remains unchanged and when the measured magnetic field $H_{x}$ is constant, the time difference ΔT is inversely proportional to the excitation amplitude ${\;H}_{m}$ and frequency f. As shown in Figure 12 and Figure 13, the sensitivity S is inversely proportional to both the excitation amplitude $H_{m}$ and frequency f.

### 2.5. Output Model Under Sawtooth Wave Excitation

When using a sawtooth wave magnetic field for excitation, assuming the excitation period is $T_{e}$, the slope of the sawtooth wave’s sloping edge is ±a, and *b* is the intercept (constant), the expression is given by Equation (17):

(17) $$H_{e}(t)=-at+b\;\;\;\;NT_{e}<t<NT_{e}+T_{e}$$

When the total magnetic field strength *H* reaches the positive and negative saturation states of the magnetic core at $t_{1}$, $t_{2}$, and $t_{3}$, the relationship between the excitation magnetic field $H_{e}(t)$, the measured magnetic field $H_{x}$, and the coercivity $H_{c}$ is given by Equation (18):

(18) $$\left\{\begin{array}{l} t_{1}:H_{x}+(-at_{1}+b)=H_{c}\\ t_{2}:H_{x}-(-a(t_{2}-\frac{T_{e}}{2})+b)=-H_{c}\\ t_{3}:t_{3}=t_{1}+T_{e} \end{array}\right.$$

It can be derived from the above equation that:

(19) $$\left\{\begin{array}{l} t_{1}=\frac{H_{x}-H_{c}+b}{a}\\ t_{2}=\frac{-H_{x}-H_{c}+b+\frac{{aT}_{e}}{2}}{a}\;\\ t_{3}=t_{1}+T_{e} \end{array}\right.$$

When the magnetic field strength reaches the saturation state of the magnetic core at $t_{1}$, $t_{2}$, and $t_{3}$, the time interval between the pulses of the induced signal within one period $T_{e}$ is given by Equation (20):

(20) $$\left\{\begin{array}{c} T^{+}=t_{2}-t_{1}=\frac{-{2H}_{x}+\frac{{aT}_{e}}{2}}{a}\\ T^{-}=t_{3}-t_{2}=\frac{{2H}_{x}-\frac{{aT}_{e}}{2}}{a}+T_{e} \end{array}\right.$$

Under sawtooth magnetic field excitation, the relationship between the output time difference ΔT of the RTD-fluxgate sensor and the measured magnetic field $H_{x}\;$ is given by Equation (21):

(21) $$\Delta T=T^{+}-T^{-}=\frac{{2H}_{x}}{a}-\left(-\frac{{2H}_{x}}{a}\right)=\frac{{4H}_{x}}{a}=\frac{{4H}_{x}}{{2H}_{m}f}=\frac{{2H}_{x}}{H_{m}f}$$

The expression for the sensitivity S of the RTD-fluxgate sensor is as follows:

(22) $$S=\frac{\partial \Delta T}{\partial H_{x}}=\frac{4}{a}=\frac{2}{H_{m}f}$$

**Figure 14:**
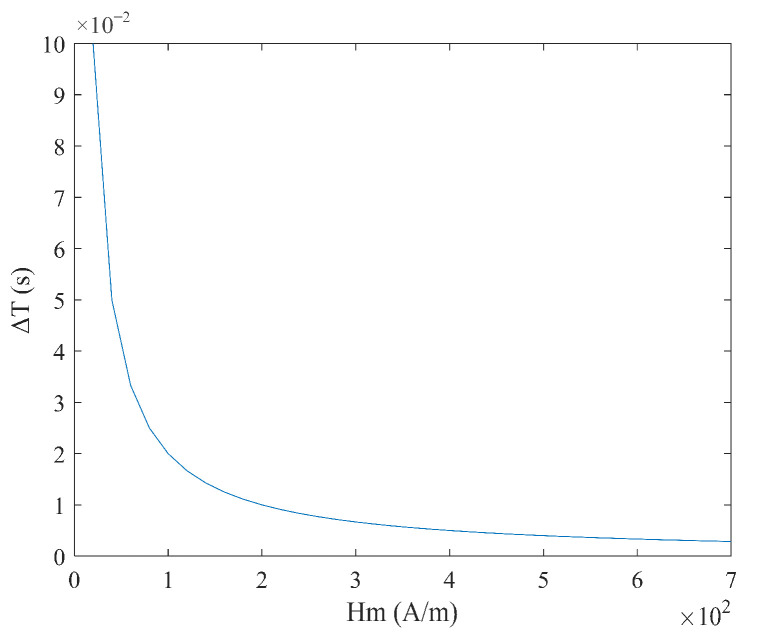
Relationship between time difference ΔT and excitation amplitude${\;\;H}_{m}$ under sawtooth wave excitation.

**Figure 15:**
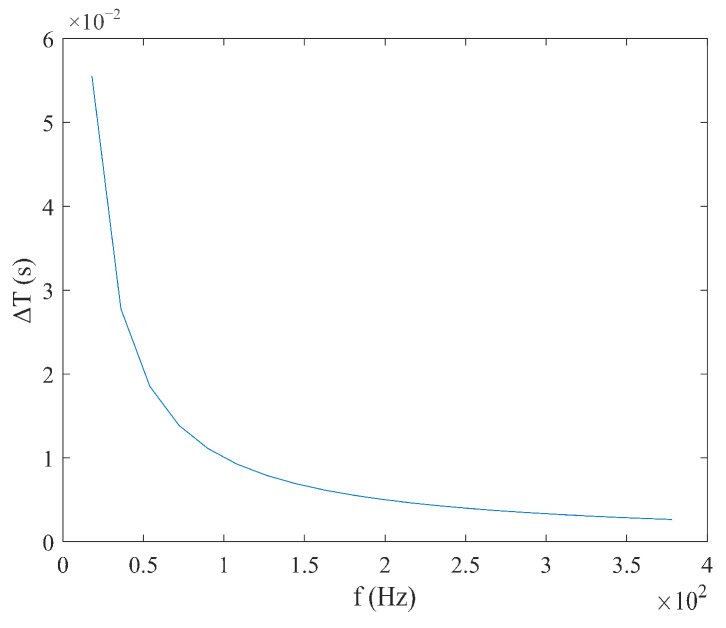
Relationship between time difference ΔT and frequency f under sawtooth wave excitation.

**Figure 16:**
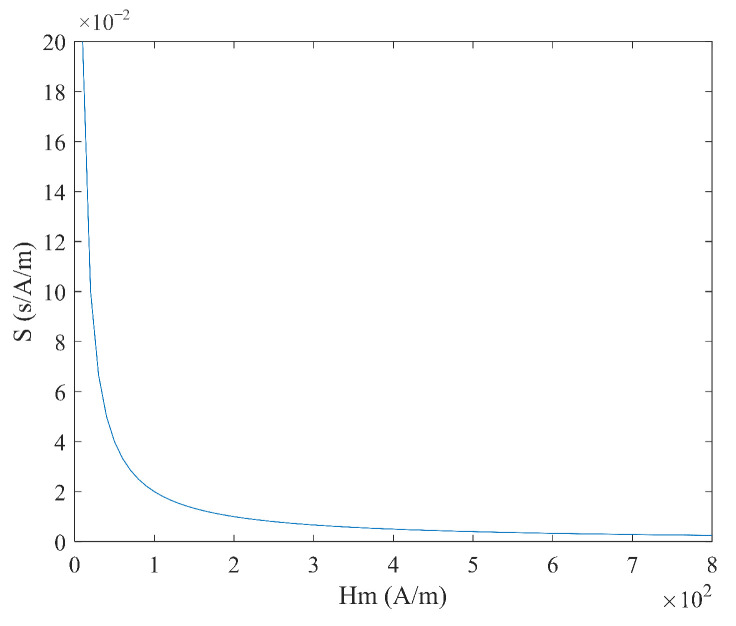
Relationship between sensitivity S and excitation amplitude $H_{m}$ under sawtooth wave excitation.

**Figure 17:**
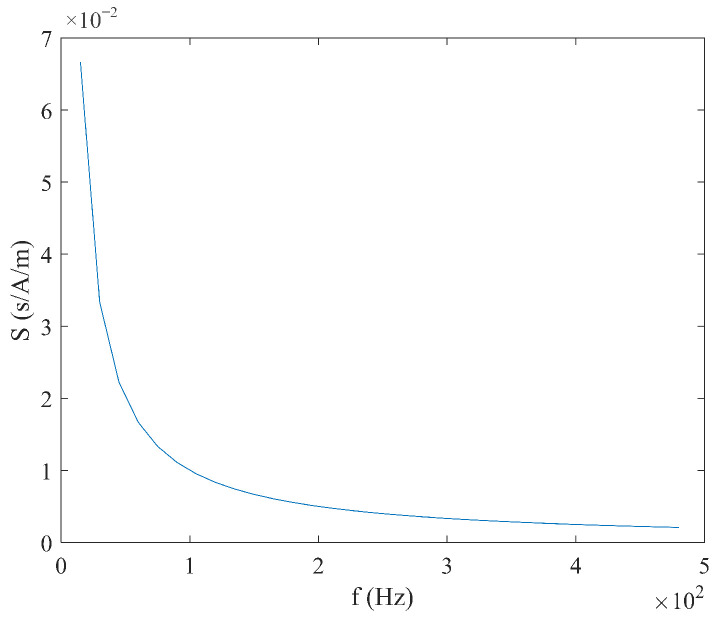
Relationship between sensitivity S and frequency f under sawtooth wave excitation.

As shown in Equation (21), when the sawtooth wave magnetic field is used for excitation, the coercivity $H_{c}$ does not need to be considered, and the time difference ΔT has a linear relationship with the measured magnetic field $H_{x}$, with a simple expression. When the measured magnetic field $H_{x}$ is constant, the relationship between the time difference ΔT and the excitation amplitude $H_{m}$ and frequency f is shown in Figure 14 and Figure 15, where the smaller the excitation amplitude ${\;H}_{m}$ and frequency f, the larger the time difference ΔT. As shown in Equation (22), under sawtooth wave magnetic field excitation, the sensitivity S is independent of the measured magnetic field${\;H}_{x}$ and is determined only by the excitation amplitude $H_{m}$ and frequency f. As shown in Figure 16 and Figure 17, the sensitivity S is inversely proportional to the excitation amplitude${\;H}_{m}$ and frequency f.

## 3. Simulation Results Analysis

**Figure 18:**
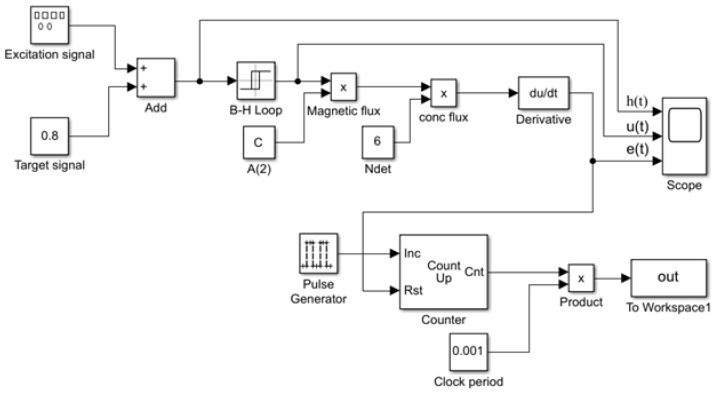
Block diagram of the simulation model for the working principle of the RTD-fluxgate sensor.

Based on the detection principle of the RTD-fluxgate sensor, a simulation model of the sensor’s working principle was established using MATLAB Simulink R2021a, as shown in Figure 18.

**Figure 19:**
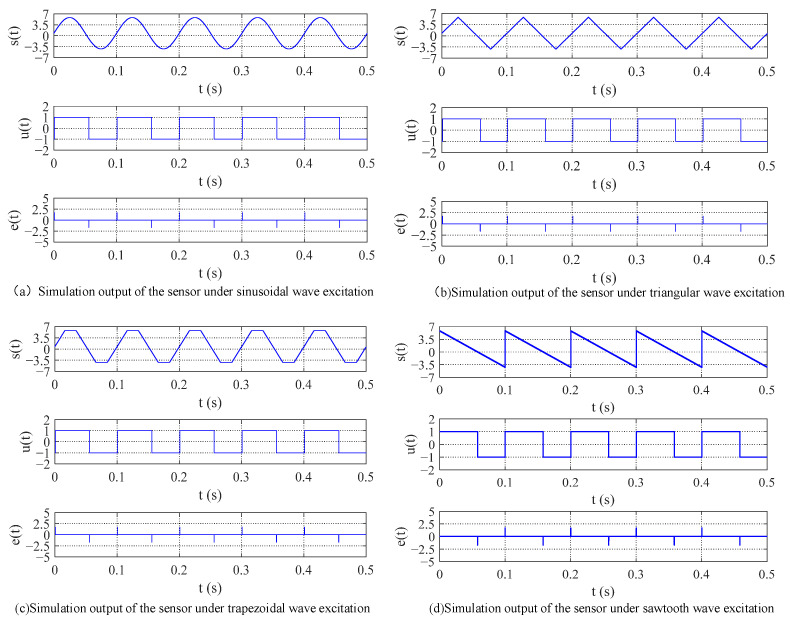
Simulation output of the RTD-fluxgate sensor under different waveform excitations.

In the simulation model, the excitation magnetic field was generated by a waveform generator and could be set to different waveforms. $h\left(t\right)$ represents the axial total magnetic field strength of the sensing unit’s core, equivalent to the sum of the excitation magnetic field $H_{e}(t)$ and the measured magnetic field ${\;H}_{x}$. An ideal hysteresis loop model was simulated using a Schmitt trigger, with the switching threshold set to the coercivity $H_{c}$ =  ±1 A/m. $u\left(t\right)$ corresponded to the magnetic flux density $B\left(t\right)$, and the induced voltage $e\left(t\right)$ was composed of a multiplication module and a du/dt module. When using sinusoidal, triangular, trapezoidal, and sawtooth wave magnetic field excitations with the same frequency and amplitude and with the measured magnetic field ${\;H}_{x}$ = 0.8 A/m, the simulation results of the residence time difference fluxgate sensor are shown in Figure 19.

**Figure 20:**
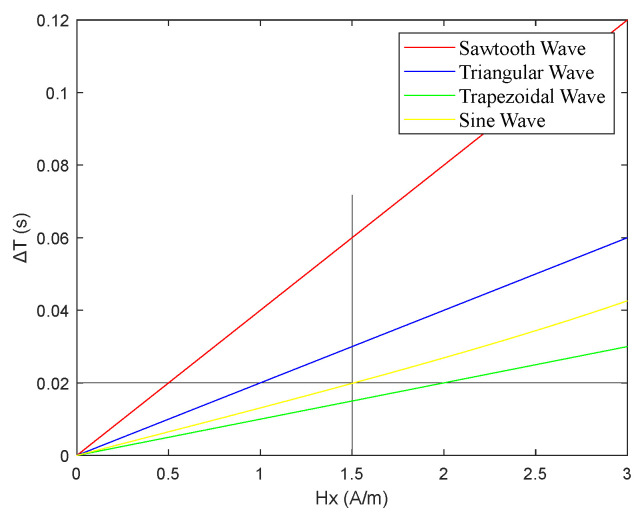
Relationship between the time difference ΔT and the measured magnetic field ${\;H}_{x}$ under different waveform excitations with the same frequency and amplitude.

From Figure 11, it can be preliminarily observed that among the pulse peaks of the induced voltage $e\left(t\right)\;$ generated by the four excitation waveforms, the interval between the pulse peaks of the sawtooth wave was the largest. Using MATLAB, the simulation results for the relationship between the output time difference ΔT of the RTD-fluxgate sensor and the measured magnetic field ${\;H}_{x}$ under sawtooth, triangular, trapezoidal, and sinusoidal magnetic field excitations with the same frequency and amplitude are shown in Figure 20.

From Figure 20, it can be observed that when using different waveform excitations with the same frequency and amplitude, the time difference ΔT with sawtooth wave excitation was larger, compared to triangular, trapezoidal, and sine wave excitations.

To better illustrate the advantages of sawtooth wave excitation in terms of sensitivity under the same frequency and amplitude, the simulation model of the RTD-fluxgate sensor shown in Figure 18 was used to perform comparative simulations under the following three conditions.

**Figure 21:**
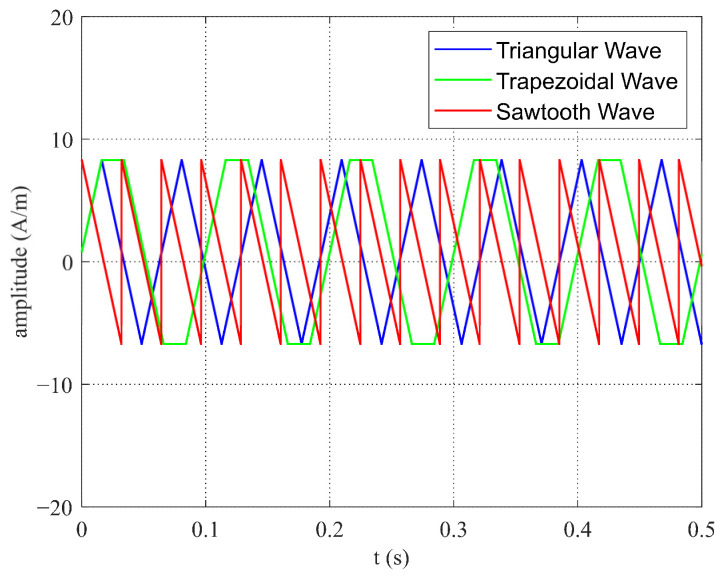
Excitation waveforms of sawtooth, triangular, and trapezoidal waves with identical amplitude and slope.

**Figure 22:**
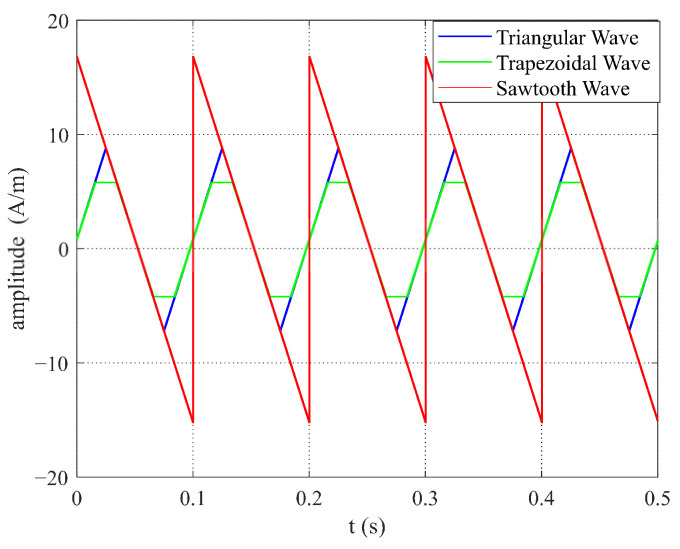
Excitation diagrams of sawtooth, triangular, and trapezoidal waves with the same frequency and slope.

**Figure 23:**
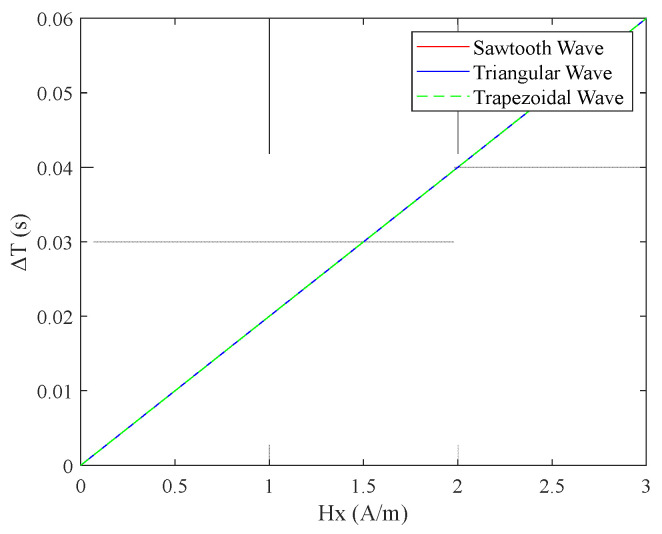
Relationship between time difference ΔT and the measured magnetic field $H_{x}$.

**Figure 24:**
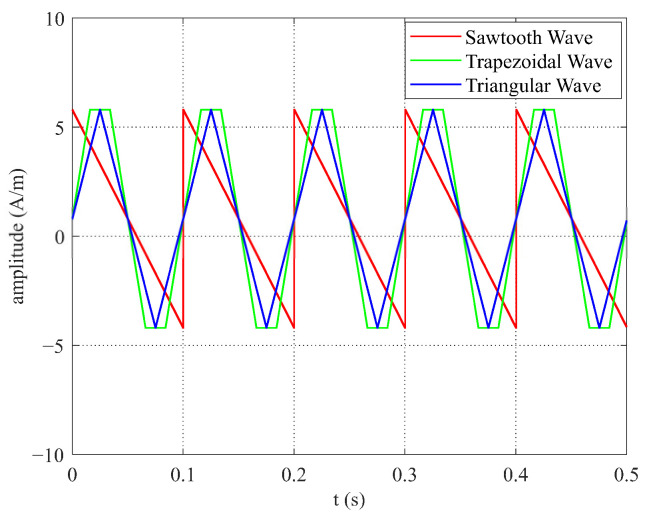
Excitation diagrams for sawtooth, triangular, and trapezoidal waves with equal frequency and amplitude.

**Figure 25:**
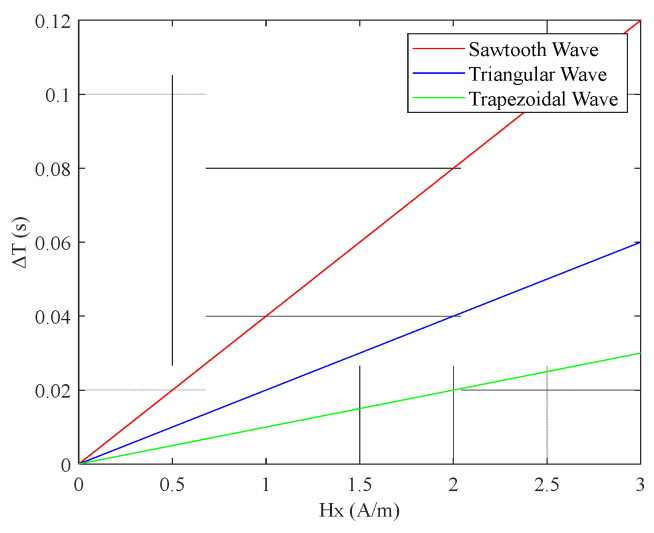
Relationship between time difference ΔT and the measured magnetic field ${\;H}_{x}$.

(1) When the amplitude and slope of the sawtooth, trapezoidal, and triangular wave magnetic field excitations were the same, the frequency of the sawtooth wave was the highest, as shown in Figure 21. With sawtooth wave excitation, the time during which the output pulse wave of the shaped, induced signal remained within the duty cycle was shorter, compared to triangular and trapezoidal wave excitations. Therefore, the times for the sensing unit’s core to reach positive saturation $T^{+}$ and negative saturation ${\;T}^{-}$ were also shorter with sawtooth wave excitation, as indicated by the simulation data in Table 1. Additionally, the energy dissipated by the sensing unit’s core during one complete magnetization cycle was related to the excitation frequency. Since the frequency of sawtooth wave excitation was higher, it led to increased system power consumption. Therefore, it is important to choose the excitation waveform and frequency wisely to ensure optimal sensor performance and to avoid negative impacts.

(2) When the frequency and slope of the sawtooth, trapezoidal, and triangular wave excitations were the same, the amplitude of the sawtooth wave excitation was higher, as shown in Figure 22. Figure 23 indicates that the relationship between the time difference ΔT and the measured magnetic field $H_{x}$ was consistent for the three excitation waveforms. The time difference ΔT of the fluxgate sensor was equal, and the variation in ΔT with respect to each waveform matched the expressions (Equations (9), (15) and (21)) when the excitation slopes were equal, as well as the simulation data shown in Table 2. When the sawtooth wave excitation magnetic field reached the saturation state of the sensor’s sensitive unit core, the amplitude of the excitation magnetic field continued to increase, which increased the sensor’s power consumption. Therefore, it is also important to carefully select the excitation waveform and amplitude to avoid negative impacts on the sensor’s performance.

(3) When the frequency and amplitude of the excitation magnetic fields for the sawtooth, trapezoidal, and triangular waves were equal, the sawtooth wave exhibited the smallest slope, as shown in Figure 24. As illustrated in Figure 25, among the three excitation waveforms, the sawtooth wave resulted in the largest time difference ΔT. According to the simulation data in Table 3, under the same frequency and amplitude conditions, the time difference ΔT for the sawtooth wave excitation was 2 times larger than that for the triangular wave and 3.3 times larger than that for the trapezoidal wave. The simulation data are consistent with the relationships among the time-difference expressions for each waveform under these conditions. According to the sensitivity expression for the RTD-fluxgate sensor, $S=\frac{\partial \Delta T}{\partial H_{x}}$, a larger time difference ΔT led to a higher sensitivity S. In summary, using the sawtooth wave as the excitation waveform under these conditions significantly enhances the sensor’s sensitivity.

## 4. Conclusions and Discussion

When using conventional waveform excitation, issues arise, such as the influence of coercive force on the time difference expression and limitations in sensitivity improvement. Therefore, this study proposed the use of sawtooth wave magnetic field excitation, deriving expressions for the time difference and sensitivity and comparing the output characteristics under different waveform excitations. Simulation results indicated that the time difference expression for sawtooth wave excitation does not require the consideration of coercive force. The time difference ΔT was 2 times that of triangular wave excitation and 3.3 times that of trapezoidal wave excitation, significantly improving the sensor’s sensitivity. This paper is expected to play a crucial role in the field of UAV aeromagnetic gradient detection and to promote the development of this area.

Finally, it must be emphasized that the proposed sawtooth wave excitation method has established a solid foundation for advancing UAV-based aeromagnetic detection through comprehensive theoretical analysis and simulation studies. Although the simulation results demonstrated significant performance improvements, these findings have yet to be validated through experimental testing with actual RTD-fluxgate sensors. We acknowledge this limitation in our study and will prioritize systematic experimental investigations as a key focus of our future research efforts.

## Figures and Tables

**Table 1:** Simulation data for various waveforms with the same amplitude and slope.

	T^+^	T^−^	ΔT
Triangular Wave	0.0356	0.0289	0.0067
Trapezoidal Wave	0.0534	0.0467	0.0067
Sawtooth Wave	0.0199	0.0122	0.0077

**Table 2:** Simulation data for different waveforms with the same frequency and slope.

	T^+^	T^−^	ΔT
Triangular Wave	0.055	0.0451	0.0099
Trapezoidal Wave	0.0551	0.0445	0.0101
Sawtooth Wave	0.0558	0.0443	0.0115

**Table 3:** Simulation data for different waveforms with the same frequency and amplitude.

	T^+^	T^−^	ΔT
Triangular Wave	0.058	0.0421	0.0159
Trapezoidal Wave	0.0551	0.045	0.0101
Sawtooth Wave	0.068	0.0321	0.0359

## Data Availability

Data are contained within the article.
